# Optofluidic Force Induction Meets Raman Spectroscopy and Inductively Coupled Plasma-Mass Spectrometry: A New Hyphenated Technique for Comprehensive and Complementary Characterizations of Single Particles

**DOI:** 10.1021/acs.analchem.3c04657

**Published:** 2024-05-14

**Authors:** Christian Neuper, Marko Šimić, Thomas E. Lockwood, Raquel Gonzalez de Vega, Ulrich Hohenester, Harald Fitzek, Lukas Schlatt, Christian Hill, David Clases

**Affiliations:** † Brave Analytics GmbH, 8010 Graz, Austria; ‡ Gottfried Schatz Research Center, Medical Physics and of Biophysics, Medical University of Graz, 8010 Graz, Austria; § Institute of Physics, University of Graz, 8010 Graz, Austria; ∥ University of Technology Sydney, 2007 Ultimo, Australia; ⊥ Institute of Chemistry, University of Graz, 8010 Graz, Austria; # Nu Instruments, LL13 9XS Wrexham, United Kingdom; ∇ Graz Centre for Electron Microscopy, 8010 Graz, Austria

## Abstract

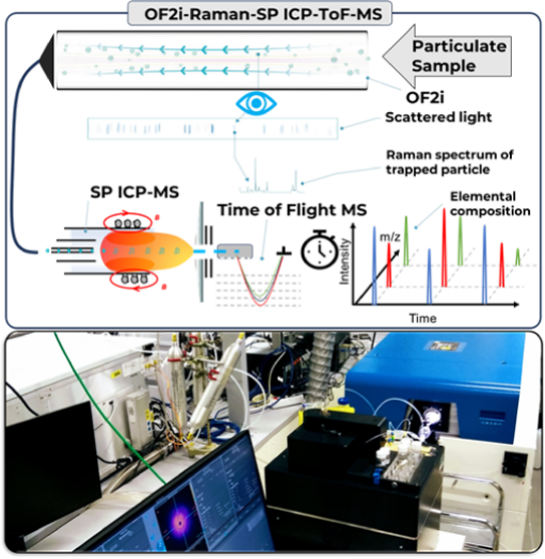

Nanoparticles are produced at accelerating rates, are increasingly integrated into scientific and industrial applications, and are widely discharged into the environment. Analytical techniques are required to characterize parameters such as particle number concentrations, mass and size distributions, molecular and elemental compositions, and particle stability. This is not only relevant to investigate their utility for various industrial or medical applications and for controlling the manufacturing processes but also to assess toxicity and environmental fate. Different analytical strategies aim to characterize certain facets of particles but are difficult to combine to retrieve relevant parameters coherently and to provide a more comprehensive picture. In this work, we demonstrate the first online hyphenation of optofluidic force induction (OF2i) with Raman spectroscopy and inductively coupled plasma-time-of-flight-mass spectrometry (ICP-TOFMS) to harness their complementary technology-specific advantages and to promote comprehensive particle characterizations. We optically trapped individual particles on a weakly focused vortex laser beam by aligning a microfluidic flow antiparallelly to the laser propagation direction. The position of particles in this optical trap depended on the hydrodynamic diameter and therefore enabled size calibration as well as matrix elimination. Additionally, laser light scattered on particles was analyzed in a single particle (SP) Raman spectroscopy setup for the identification of particulate species and phases. Finally, particles were characterized regarding elemental composition and their distributions in mass and size using SP ICP-TOFMS. In a proof of concept, we analyzed polystyrene-based microplastic and TiO_2_ nanoparticles and demonstrated the opportunities provided through the coupling of OF2i with SP Raman and SP ICP-TOFMS.

## Introduction

Nanomaterials are being developed at an increasing rate and are driving technological advances in many fields of science.^1−3^ Any manufacturing and application of nanomaterials, related process controls, and investigations of intrinsic properties and particle fate are dependent on suitable analytical technologies, which enable the determination of relevant physical and chemical parameters.^3,4^ These comprise size and mass distributions, particle number concentrations, stability, and the elemental and molecular compositions. Since Faraday’s seminal work on particles marking the beginning of modern colloidal science,^5^ it took more than a century until analytical technologies gained at least partial capabilities to assess particle-specific parameters quantitatively. Modern technologies address some initial shortcomings and provide new perspectives. For example, the surface potential (ζ-potential) may be evaluated by measuring particle electrophoretic mobility,^6^ the hydrodynamic diameter can be estimated via dynamic light scattering (DLS) or nanoparticle tracking analysis (NTA),^6,7^ and compositions can be studied using spectroscopic techniques [e.g., energy dispersive X-ray (EDX) analysis, Raman scattering].^8,9^ While these techniques may describe certain properties of particles, they are not applicable to decipher properties coherently and fail to provide a comprehensive perspective.

The introduction of inductively coupled plasma-mass spectrometry (ICP-MS) set new benchmarks for the characterization of nanomaterials.^10^ While in its conventional setup, it allowed the analysis of the overall elemental content in suspensions,^11^ its single particle mode (SP) surpassed this capability and enabled the counting of individual particles and the establishment of models on particle number concentrations, size, and mass distributions as well as on particle stability.^3,12^ SP ICP-MS was further improved through the maturation of time-of-flight technology (ICP-TOFMS), which enabled the additional establishment of models on elemental and isotopic compositions in single particles as well as non-target screenings.^13,14^

For the construction of size and mass distribution models, SP ICP-MS relies on parameters that often are only approximated. For example, models assume that all particles are perfectly spherical and that phases, density, and elemental mass fractions are known and consistent throughout particles.^15^ Furthermore, SP ICP-MS does not consider particles or particle fractions consisting of incompatible elements or which are below the size detection limit. For most elements, the latter is in the low- or mid-nanometer range^16^ but can increase up to the micrometer scale when, for example, targeting C in microplastics.^17^ When analyzing dispersions with heterogeneous and/or unknown particles, model assumptions are often unrealistic, and it is not known how many particles are missed and misinterpreted. To consider otherwise missed particles as well as to enhance characterizations, complementary techniques are required. One promising method to achieve such a complementary perspective is optofluidic force induction (OF2i), which was recently introduced as an online tool for nanoparticle characterization.^23^ This technique is based on the optical counting and manipulation of individually trapped particles and provides number-based size distributions, as well as estimations of number concentrations. Advancing on the concept of optical tweezers^18−20^ and two-dimensional (2D) optical traps,^21^ OF2i uses a weakly focused vortex beam with a wavelength of 532 nm, which is aligned in parallel to the microfluidic flow direction to observe single particle acceleration for the measurement of size distributions as well as number concentrations.^22−24^

In this work, we propose an antiparallel alignment of the laser and flow direction to trap particles on the vortex beam. This enabled the spatial separation of differently sized particles and allowed an optical chromatography setup.^21^ This was useful to achieve a matrix separation and to trap particles continuously according to preselected size-based criteria. The trapping of particles in the vortex beam also enabled the direct observation of Raman scattering to deduce chemical species and mineral phases contained in the individual particles. Finally, OF2i can be coupled to SP ICP-TOFMS to characterize element compositions of released particles. This was showcased for the examples of polystyrene-based microplastics and TiO_2_ particles, which are ubiquitously found throughout the environment and which are discharged through anthropogenic sources.^25,26^

## Materials and Methods

### Consumables and Sample Preparation

Polystyrene (PS)-based microplastic particles in water were obtained from Sigma-Aldrich (10% solid content, density of 1.05 g/cm^3^, cross-linking degree of 2%) at 5 μm (±0.1 μm) and from Thermo Scientific (3000 Series NIST traceable Nanosphere Size Standards, ∼1% solid content, density of 1.05 g/cm^3^) with nominal diameters of 303 nm (±6 nm), 401 nm (±6 nm), and 600 nm (±9 nm). For dilutions, ultrapure water was obtained from a Merck Millipore system (18.2 MΩ cm, Bedford). TiO_2_ particles were obtained with a mean diameter of 21 nm (≥99.5%, primary size, Sigma-Aldrich) but showed pronounced agglomeration when dispersed in ultrapure water. pH and buffer concentrations were adapted to increase the stability of the suspension. The determination of transport efficiency in SP ICP-TOFMS was carried out, monitoring a core–shell particle containing Au (50 nm core) and Ag (15 nm shell thickness) and analyzing ionic standards containing 10 ng/g Au and Ag. Ultrapure water was used as a blank for background subtraction. The mass calibration of TiO_2_ particles via SP ICP-TOFMS used an ionic 10 ng/g Ti standard, and microplastic calibration was carried out with a 4 μm PS reference particle (10% solids, 1.05 g/cm^3^ and a cross-linking degree of 2%, Sigma-Aldrich).

### Instrumentation

The OF2i instrument (BRAVE B-Curious) was built on a 2D optical trap in a cylindrically shaped microfluidic flow channel with an inner diameter of 1.3 mm. A linearly polarized laser beam was generated by a 532 nm CW DPSS laser with a maximum power of 2 W (Laser Quantum, GEM532). Then, beam alignment was performed using two mirrors and a 5× beam expander. Using a zero-order vortex half-wave plate (*q* = 1), an azimuthally polarized Laguerre–Gaussian laser mode with topological charge *m* = 2 was generated and focused into the measurement cell. A microfluidic pump was connected to deliver flow rates between 1 and 300 μL/min antiparallel to the vortex beam. Light scattered by trapped particles was magnified and recorded by using an ultramicroscope setup. For the extraction of Raman spectra, the ultramicroscope setup was modified and extended by optical alignment elements, e.g., cylindrical lenses and a prism. Similarly, the measured Raman signal was then recorded by using a CMOS camera.

The ICP-TOFMS system (Nu Instruments, Wrexham, U.K.) was equipped with an SC (single cell) introduction kit (Elemental Scientific, Omaha) to enhance transport efficiency. The SC kit was operated with a nebulizer gas flow and makeup gas flow of 0.45 and 0.60 L/min, respectively. The transport efficiency was analyzed in SP ICP-TOFMS using particle standards with known size and was determined to be 66%.

**Figure 1:**
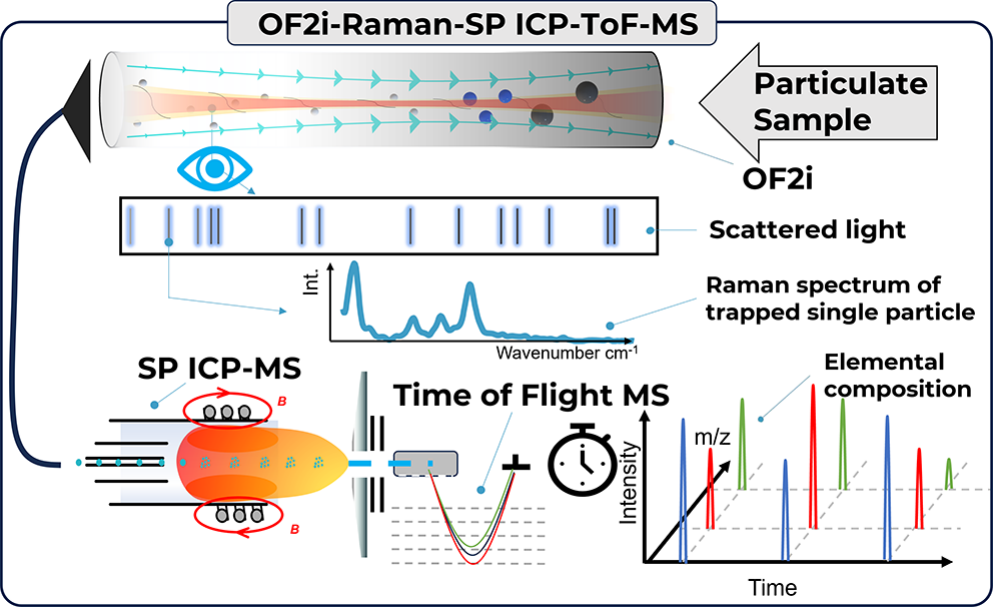
Schematic overview of techniques combined in the framework of this study to improve the characterization of individual particles.

It is possible to establish a multitechnique platform that features OF2i, SP Raman, and SP ICP-TOFMS as indicated in Figure 1. In this study, the first SP Raman prototype was built and coupled with OF2i in laboratory with an adequate laser safety protocol in place. In parallel, OF2i was coupled with SP ICP-TOF-MS in a mass spectrometry lab. As such, OF2i-SP Raman and OF2i-SP ICP-TOFMS analyses were conducted separately. However, the implementation of a Raman module into the OF2i-workflow does not impact the coupling of OF2i and SP ICP-TOFMS in any regard and the combination of all three techniques into one platform is possible.

More information for the coupling of OF2i with SP Raman and SP ICP-TOFMS is available in the Supporting Information (SI).

### Software and Data Processing

The OF2i instrument was operated with the BRAVE Analytics proprietary software suite HANS, providing modulation of laser power, flow rate, and direction. A live video feed was recorded at 200 fps and saved for subsequent analysis. The OF2i-Raman data feed was recorded at a rate of 7.7 fps and evaluated using in-house-developed MATLAB routines.

TOF data was acquired with CODAC software (Nu Instruments), and spectra were recorded every 0.024 ms. Two mass spectra were binned before and after baseline subtraction, respectively, and the resulting spectra were stored at 10.4 kHz.

SP ICP-TOFMS data sets were analyzed using a homemade software named “SPCal” (Version 1.1.2),^15^ which was expanded to analyze TOF-based data. To differentiate between noise and SP signals from microplastics, a compound Poisson distribution^27,28^ was approximated using a log–normal approximation method factoring in the number of binned spectra and the single ion area signal distribution of the analogue-to-digital-converter.^14^ For microplastics, an α value of 1 × 10^–6^ was used as the decision limit. The compound Poisson thresholds for ^12^C and ^13^C were 18.6 and 7.5 cts, respectively. Due to a high background abundance of (small) TiO_2_ particles, a manual decision limit was set to 100 nm to only consider large TiO_2_ particles trapped previously in OF2i and to omit signals from smaller TiO_2_ contaminants.

## Results and Discussion

### Optofluidic Force Induction

In previous work, the setup of OF2i involved the parallel alignment of fluidic and optical forces. This enabled a trapping of particles via gradient forces in the weakly focused vortex beam and a subsequent acceleration via scattering forces in the propagation direction.^23^ After trapping, the particles moved along the intensity maxima of the laser beam with a propagation distance *z* and velocity *v* in the presence of optical scattering forces and fluidic forces. As the optical force depended on particle radius, the change of velocity could be translated into a particle size and the observation of several particles enabled the investigation of a particle size distribution.^22,23^

**Figure 2:**
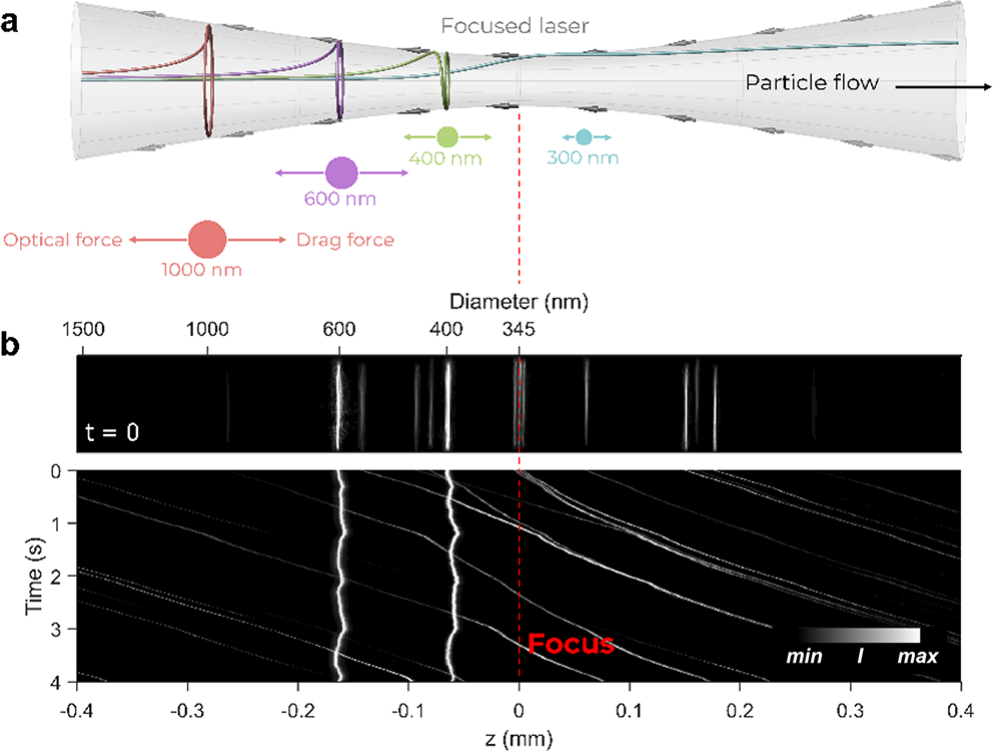
Differently sized particles have size- and refractive index-specific trapping positions. Panel (a) shows the simulated trajectories along the vortex beam (gray cone) for four PS particles with diameters of 300, 400, 600, and 1000 nm. At stable trapping positions, drag and optical forces are equal and particles are orbiting at a stationary *z*_trap_-position. Particles below a tunable threshold do not have a stable position and are eliminated from the vortex beam. (b) Experimental analysis of PS particles with 303, 401, and 600 nm. Each line represents the scattered light of a particle in the vortex beam. The bottom shows the time dependency and shows that particles above 350 nm are trapped at positions predicted by the simulation in panel (a). Smaller particles were eliminated.

In this work, we aligned the laser beam antiparallelly to the fluid direction such that the optical force decelerated the particle. The particle came to a complete halt at a stable trapping position *z*_trap_ where the optical force counteracted the drag force of the fluid. Figure 2a shows simulated trajectories of PS-based plastic particles with different diameters transported by the fluid in the presence of the focused laser beam propagating from right to left. It is apparent that visible particles were first pushed toward the intensity maxima by the gradient forces and then became trapped at size-dependent positions where they continued to orbit around the optical axis due to the orbital angular momentum of the vortex beam. This momentum played a pivotal role and gave rise to the ring-shaped intensity profile in the transverse directions and allowed simultaneous trapping of several particles with identical size and with a strong suppression of mutual scatterings as will be seen later. Also, the unhindered passing of smaller particles through trapping regions was only possible with such a configuration. The minimum and maximum trappable sizes were tuned by altering fluidic flow rates as well as laser intensity. In this case, parameters were set to trap particles with diameters between 350 and 1500 nm, and particles below 350 nm continued to flow through the capillary (compare the trajectory of blue particle with *d* = 300 nm; Figure 2a). More details are available in the SI.

Simulations were subsequently compared to experimental trajectories, as shown in Figure 2b. The position of particles in a glass capillary was determined using an ultramicroscope setup to record scattered laser light.^22^ The round capillary acted as a cylindrical lens^29^ shaping the scattered light of each particle to a line. For comparison to simulated trapping positions, PS particles with nominal diameters of 303, 401, and 600 nm were analyzed at a constant flow rate of 7 μL/min and a 1 W laser beam power. From the *z*_trap_ value, particle sizes were calculated through our Mie theory model, which was presented in detail in a recent study^22^ and for which more information can be found in the SI. As the relevant dynamics of the trapping and elimination of particles took place in the z direction, consecutive video frames were stacked for a duration of 4 s along a vertical axis (Figure 2b, bottom). The resulting waterfall diagram shows that two particle entities (401 and 600 nm) were trapped at different positions *z*_trap_, which were in line with simulated positions shown in Figure 2a. PS particles with a diameter of 303 nm were not trapped and continued to propagate through the focal region of the laser beam as predicted. Minor time-dependent variations in *z*_trap_ were the result of fluctuations of the microfluidic pump.

**Figure 3:**
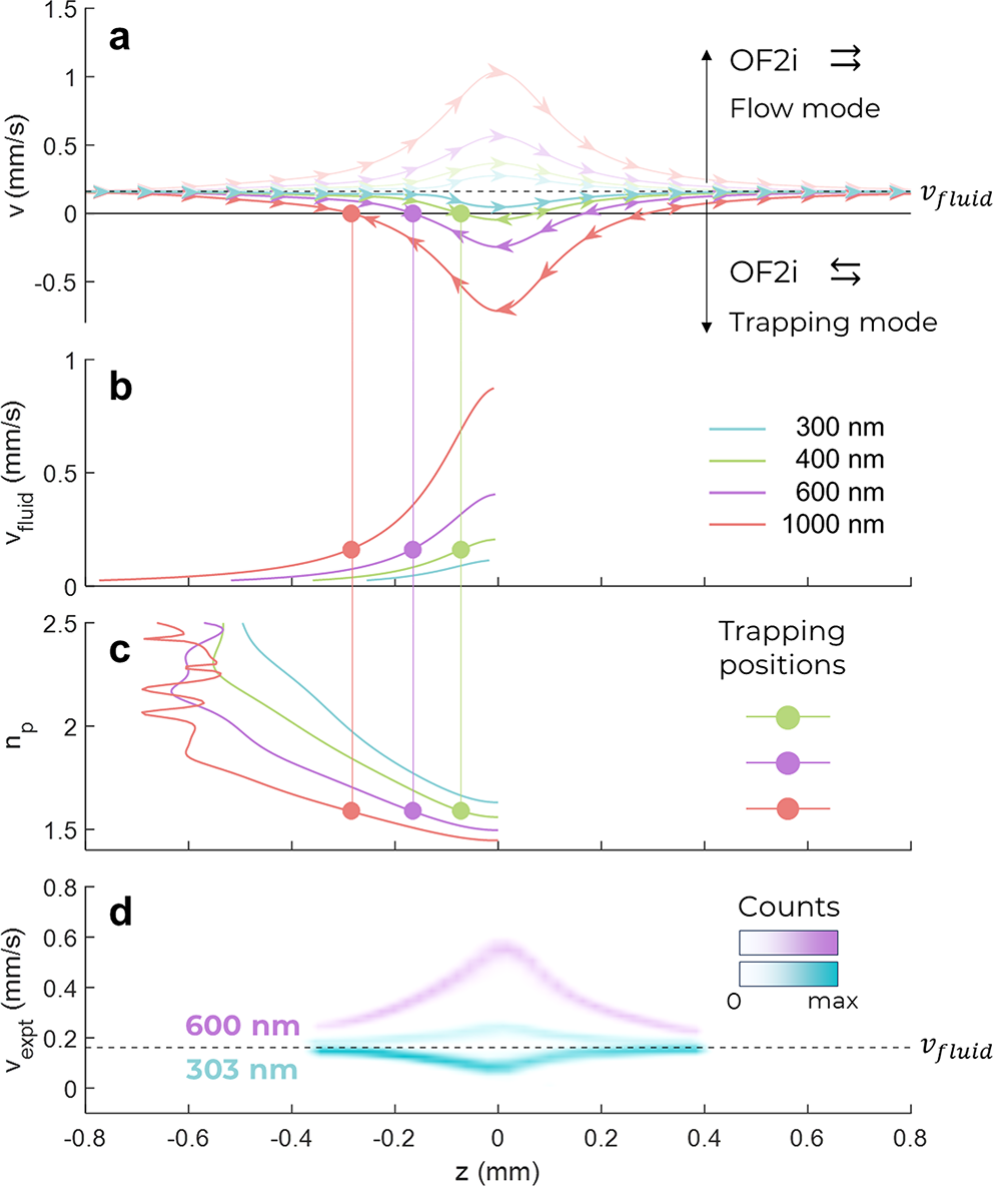
Mie theory simulations and experimental data of the OF2i particle flow and trapping mode. (a) Particles with different sizes are accelerated to different velocities *v*, which depend on the position within the vortex relative to the laser focus (*z* = 0). In flow mode, particles are accelerated in the laser propagation direction. In trapping mode, particles come to a halt at positions where optical forces and dragging forces are equal. (b) Dependency of the trapping position and flow velocity *v*_fluid_. (c) Impact of the refractive index *n*_p_ on trapping positions. (d) Histograms of experimental velocities *v*_expt_ for different z positions in the microfluidic flow cell measured from 303 and 600 nm particles in flow mode and 303 nm particles in trapping mode.

In summary, the OF2i can be operated in two modes, as demonstrated in Figure 3a. Here, simulations for both the trapping and flow modes are shown according to Mie theory.^22^ When the fluidic flow and the laser vortex beam are coaligned (“flow mode”), the acceleration of particles can be calculated into sizes. When aligning flow and laser antiparallelly (“trapping mode”) as demonstrated in the present study, particles are decelerated, and equilibrium positions on the vortex beam can be translated into particle size. The trappable size range and distinct equilibrium positions can be tuned by altering either laser power or fluid velocity as they are directly proportional to the optical and fluidic forces, respectively. The latter is simulated in Figure 3b, which shows the effect on particles when increasing *v*_fluid_. Consequently, the stable position (*z*_trap_) is shifted into a region of higher field strength (toward the laser focus) until drag and optical forces are equal again. However, optical forces are further dependent on the refractive index (*n*_p_) and therefore *z*_trap_ shifts into regions with lower laser intensity (away from the laser focus) as *n*_p_ increases. Depending on *n*_p_ and at approximate propagation distances of −0.5 mm, the onset of Mie resonances is expected and can be seen in Figure 3c. This results in a nonmonotonous dependence of *z*_trap_ and *n*_p_ and should be avoided to maintain accurate size determination for larger particles. The conditions and alignments simulated in Figure 3a for both trapping and flow modes were subsequently experimentally confirmed as shown in Figure 3d, displaying the acceleration and deacceleration of 303 and 600 nm sized PS particles in a 2D histogram, respectively.

### OF2i-Raman Spectroscopy

**Figure 4:**
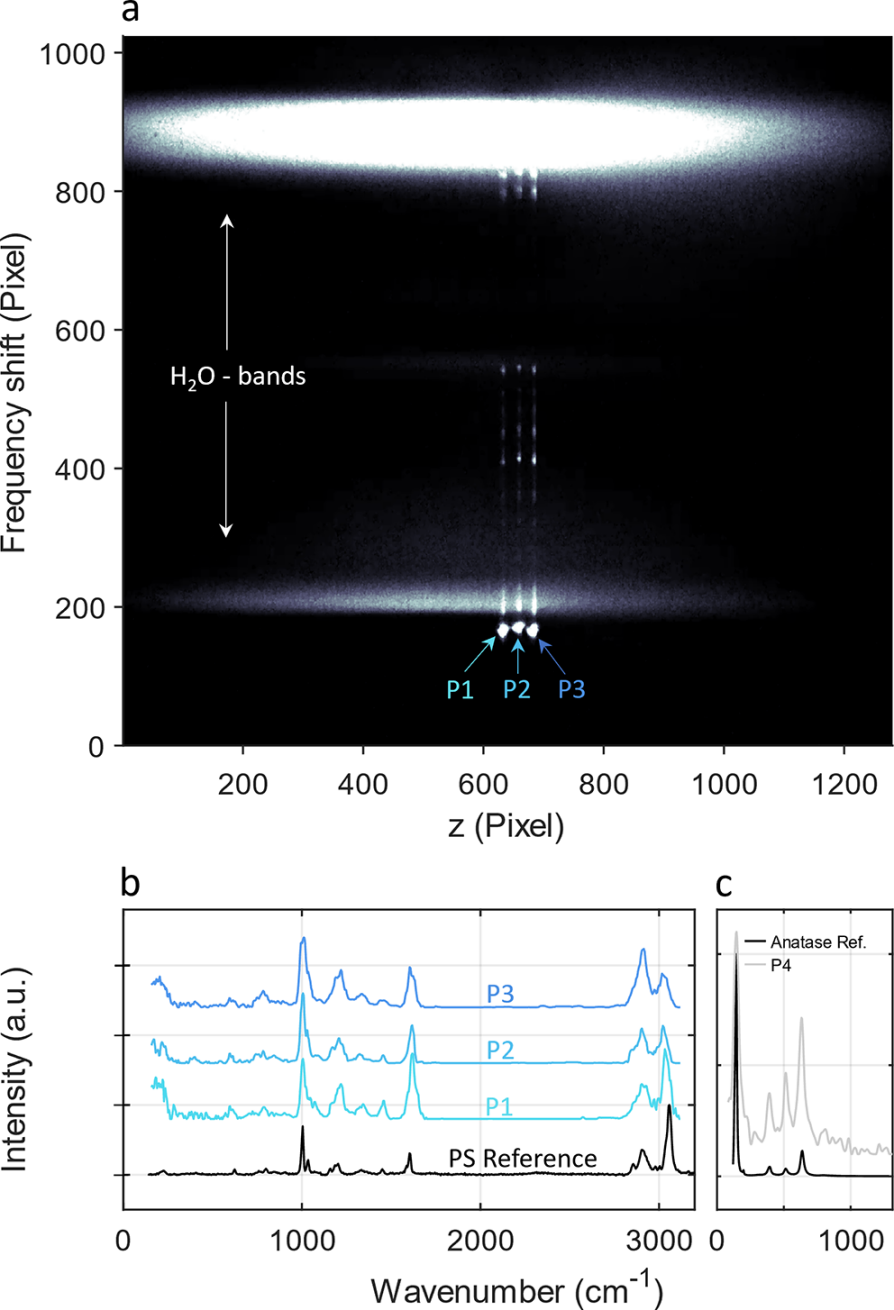
(a) Three microplastic particles were trapped in OF2i. Light scattered off particles was analyzed in an in-built Raman spectroscopy approach to identify contained species. (b) The experimental Raman spectra were compared against a reference spectrum and allowed a direct identification of microplastics as PS particles. Panel (c) shows an example where TiO_2_ was analyzed (see the SI).

The trapping mode has conspicuous advantages for certain applications such as online hyphenation to MS. The trapping of single particles allows optical precharacterization as well as a complete matrix exchange, which is adjuvant for subsequent MS when targeting biological and environmental samples with complex matrices. Furthermore, both the modeling of sizes via OF2i and via SP ICP-MS require knowledge of compound-specific parameters for underlying calibration pathways: In OF2i, the refractive index needs to be known to ensure accurate calibration. In SP ICP-MS, particle mass estimations are performed by considering mass fractions of the analyzed element. Sizes can subsequently be calculated by consideration of particle density while assuming a spherical geometry. However, given that in SP ICP-MS particles are completely atomized in the plasma, information on shape, species, and contained phases cannot be retrieved, and models and assumptions for mass and size models are often flawed. The establishment of more accurate models is reliant upon an *a priori* optical and molecular characterization. Using OF2i in conjunction with SP ICP-TOFMS provides opportunities to characterize trapped particles prior to elemental analyses. Here, we propose OF2i-Raman spectroscopy of previously trapped single particles for accurate identification and as a useful complementary method to improve modeling as shown later. Figure 4 (top) shows the Raman signal at different axial positions (z-coordinates) for three trapped individual microplastic particles. The Raman signal (bottom) of each particle was then extracted along the vertical axis (frequency shift). Again, the axial position of particles depended on the size of trapped entities, which was approximately 5 μm in this example. Given that microplastics may consist of a vast range of different polymers, which vary in chemical and physical properties, an accurate identification of the species was required before carrying out calibration routines. A comparison of the raw spectra for the three trapped particles (P1, 2, and 3) with the reference spectra is shown in Figure 4b. An excellent agreement with the reference spectra enabled a direct identification of trapped microplastic as PS. Besides polymeric particles like plastics, OF2i-Raman is further capable of analyzing inorganic particulate entities. In a proof of concept, TiO_2_ particles were trapped and characterized regarding contained phases. Figure 4c shows the Raman spectrum of one trapped particle along with one reference spectrum (anatase) and showcases the possibility to also analyze inorganic (nano)particles. More information on the analysis of TiO_2_ particles is given in Figure S2. TiO_2_ may consist of polymorphs, such as rutile or anatase phases, which have different physicochemical properties. Densities are different for both phases (rutile: 4.24 g/cm3, anatase: 3.9 g/cm^3^), and accurate size estimations depend on the *a priori* knowledge of the particle identity. However, anatase is also known to exhibit different toxicities and degradation behaviors, which are highly relevant parameters for the evaluation of the impact and fate of particles.^30^ We compared the experimental Raman spectra of two trapped particles (Figure S2b,e) against the reference spectra of both phases (rutile: Figure S2a,d; anatase: Figure S2c,f), which enabled a clear identification of particles as anatase.

### Hyphenation of OF2i and SP ICP-TOFMS

The coupling of ICP-MS with a complementary technique is a common strategy to combine technology-specific advantages. For example, chromatography may be coupled to ICP-MS to perform speciation analysis and laser ablation-ICP-MS enables the mapping of element distributions.^31,32^ The concept of hyphenated techniques is also interesting for the characterization of nanoparticle dispersions and was previously shown, for example, by coupling size exclusion chromatography or asymmetric flow field flow fractionation with ICP-MS to enable a size separation prior to characterizations with elemental mass spectrometry.^33−36^

Given that particle sizes and numbers analyzable by OF2i and SP ICP-MS were in a comparable range suggests the hyphenation of both to improve the investigation of particle properties. In this work, we combined both techniques for the first time to perform a complementary characterization of selected materials. One limiting factor for the hyphenation was a low aerosol transport efficiency in ICP-MS, which was only 1.7% using a conventional concentric nebulizer. Aiming to trap and characterize the same particles, we employed a nebulizer with higher transport efficiency to increase aerosol transport to 66%. In this proof of concept, we selected PS and TiO_2_ as micro-/nanocontaminants, which have previously been investigated in various sample types. The optical online trapping of particles enabled the complete removal of matrix components and their replacement with ultrapure water. The option of replacing matrix components prior to SP ICP-MS analysis is a useful feature for samples with complex matrices. For example, environmental or biological matrices (e.g., seawater, wastewater, urine) are problematic in ICP-MS due to spectral interferences, signal instability, and drift effects, and the possibility to separate particles of tunable size ranges from matrix components has a high utility to improve characterizations. Furthermore, online trapping and matrix removal can reduce the ionic background levels, which improves SP ICP-MS size detection limits.

**Figure 5:**
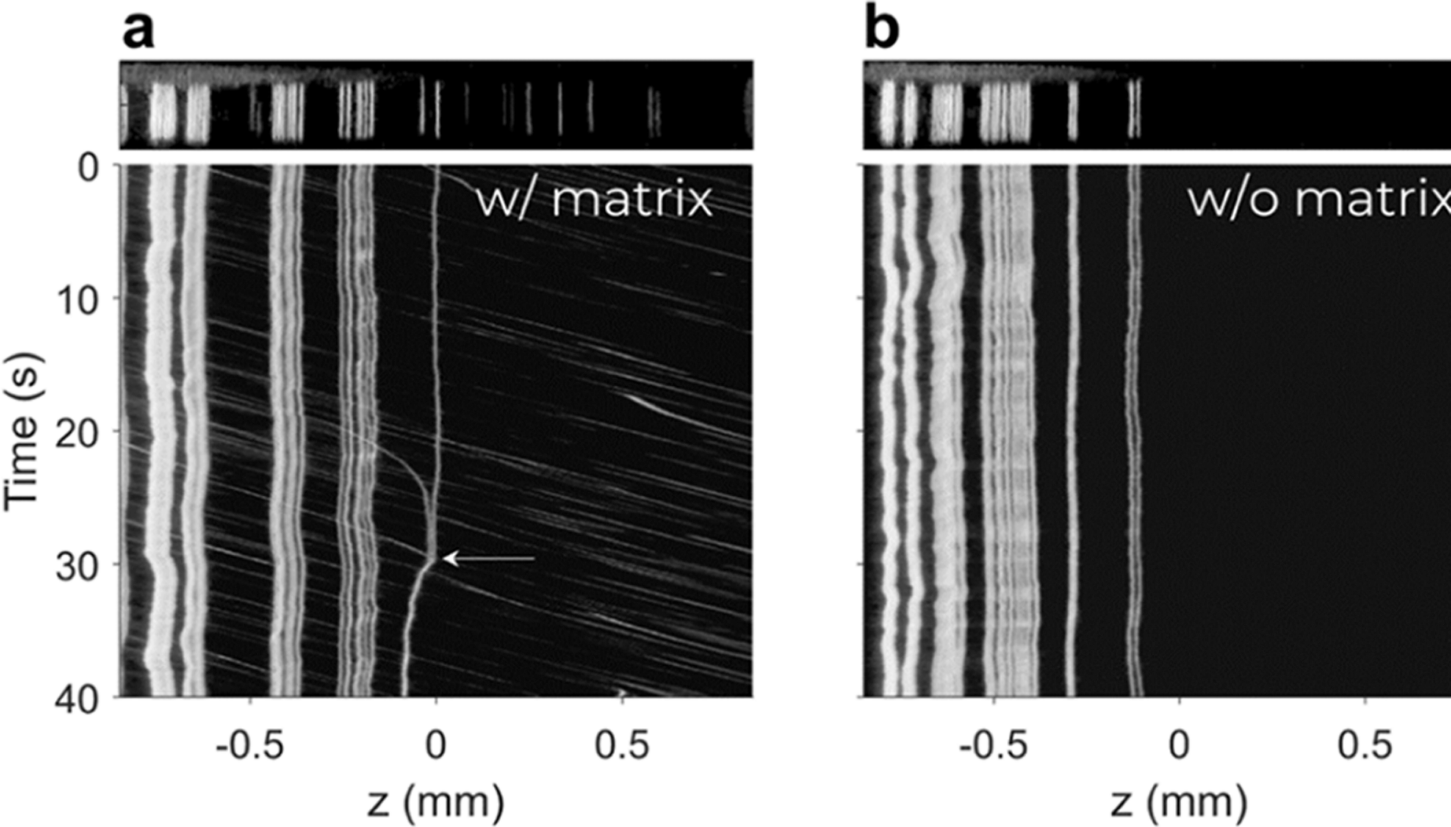
OF2i was used to trap particles above a threshold and to eliminate smaller particles as well as matrix components. (a) Initial phase of the trapping. On the top, a snapshot of the raw video material is shown. The waterfall diagram below shows several stably trapped particles over a larger period as well as the particulate matrix in the background. One particle collision/agglomeration event was observed as indicated with a white arrow at 30 s. (b) Final phase after the matrix exchange. Particles remain trapped at different positions along the laser beam, and no smaller particles and matrix components are visible any longer.

**Figure 6:**
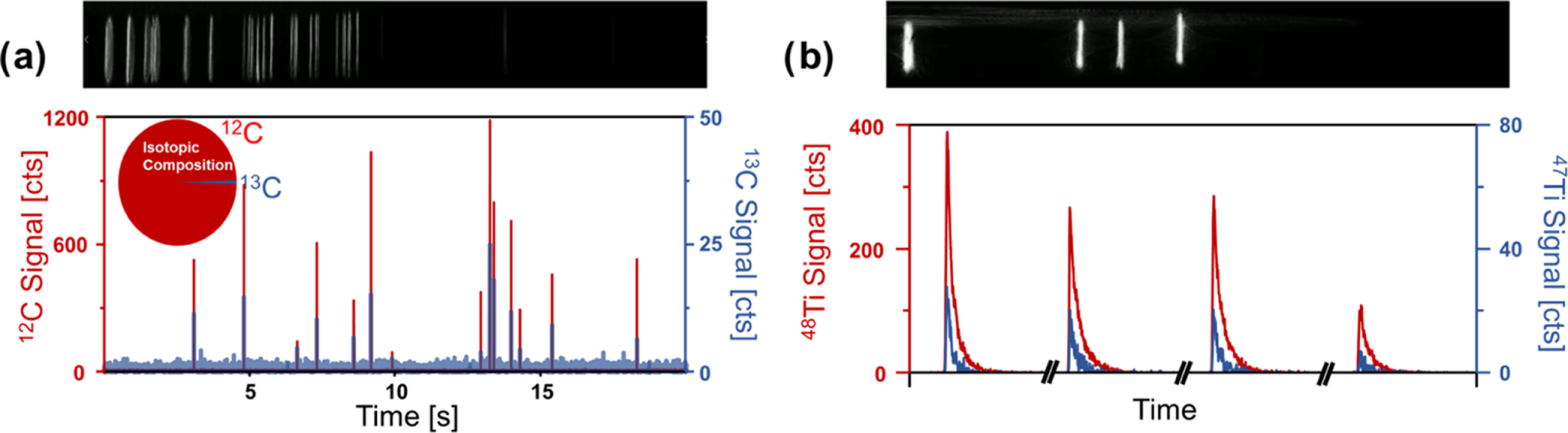
PS-based microplastics (a) and TiO_2_ particles (b) with sizes between 4 and 6 μm and above 100 nm were trapped on the vortex beam, respectively. Following trapping and matrix elimination, particles were released for SP ICP-TOFMS analysis. The application of a ToF analyzer enabled the analysis of different isotopes per particles, and single particle events were recorded with several points.

The process of matrix removal is shown in Figure 5, where the scattered light intensity of the particles is plotted logarithmically to visually enhance the weak scattering of small particles in the proximity of the vortex beam. Figure 5a shows a snapshot (top) at an early stage of the matrix removal process with the corresponding time-resolved waterfall diagram below. It is visible that some particles remained in a stable trapping position, and only small deviations were caused by fluctuations in the microfluidic pump. Particles below a threshold were not retained and were eliminated from the vortex beam over time. Following the matrix exchange, no small particles from the matrix were detected. While the vortex beam supported the trapping of particles with identical size at the same *z*-position, it was possible that particles collided to form agglomerates. This process was observed and is highlighted in Figure 5a (white arrow). The formation of a larger agglomerate was inherent with a change in the stable trapping position. The continuous trapping of particles enabled a complete matrix removal as shown in Figure 5b and was achieved in a few minutes. However, trapping and elimination experiments can easily be extended, which can be useful to trap particles at low number concentrations. Following the matrix removal and the identification of particle entities in the analyzed sample via OF2i-Raman spectroscopy, trapped particles can be released for SP ICP-TOFMS analysis. Parallelly to the abrupt release of all particles, SP ICP-TOFMS analysis was performed. Figure 6a,b shows trapped PS particles (a) and TiO_2_ particles (b) following matrix elimination. Below the optical image, time-resolved SP ICP-TOFMS data of released particles is shown. Here, PS particles were detected via ^12^C and ^13^C and compound Poisson statistics were used to differentiate particle signals from background and random noise. Fourteen of originally 21 trapped particles were recovered, which was in line with a transport efficiency of 66%. For the analysis of TiO_2_ particles, the OF2i system was set to trap particles with sizes above 100 nm and to omit smaller particles. This was useful to identify previously trapped particles in the SP ICP-TOFMS data set containing numerous signals of small TiO_2_ particles as common interference. Therefore, only trapped particles with sizes above 100 nm were considered in the SP ICP-TOFMS data set. It was possible to determine the ^12^C and ^13^C, and the ^47^Ti and ^48^Ti isotopes for the released microplastics and TiO_2_ particles, respectively. The rapid isotope acquisition of ToF-technology enabled the resolution of single particle events with several data points and the option to investigate various elements across the periodic table in the same SP event. While in this case, particle standards with known composition were analyzed, the developed techniques have a high potential for determining compositions of heterogeneous particles, the alteration of particles due to the release and/or adsorption of elements,^37^ and the origin of particles (e.g., natural vs engineered)^13^ in real-world scenarios.

### Modeling of Particle Masses and Sizes

**Figure 7:**
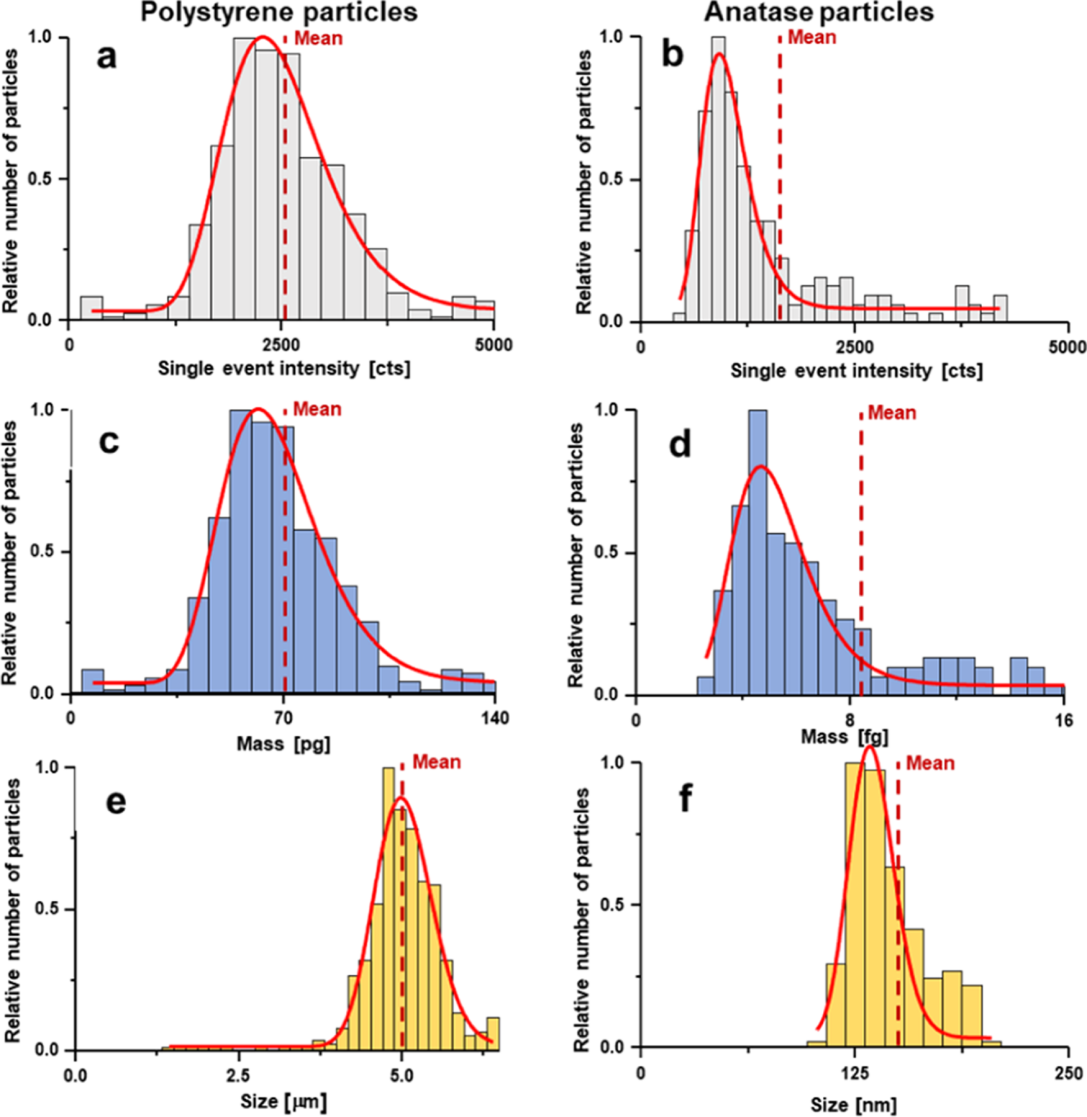
Detected PS particles (left) and anatase particles (right) were analyzed using SP ICP-TOFMS. Panels (a) and (b) show signal histograms of particles above the decision limit. Using species information obtained via Raman spectroscopy, mass distributions were modeled in panels (c) and (d). Assuming spherical geometry, particle diameters were estimated in parts (e) and (f).

While it is possible to combine OF2i, SP Raman, and SP ICP-TOFMS in one platform, OF2i-SP Raman and OF2i-SP ICP-TOFMS were coupled separately in two individual steps due to laser safety requirements. Using OF2i-SP Raman, a fraction of particles was trapped and optically characterized as a subsample beforehand to identify particle species and phases, which were parameters required for size and mass calculations. Using OF2i-SP ICP-TOFMS, it was possible to trap and release particles and to characterize the same particles that had previously been trapped, as shown in Figure 6. However, to generate representative models on mass and size distributions, a larger number of particles were required. Therefore, OF2i-Raman was used to identify the dominant particle species, and sizes and masses were calculated based on particles detected in OF2i-SP ICP-TOFMS. Figure 7a,b shows the signal histograms recorded by analyzing ^12^C and ^48^Ti, respectively. The identification as PS and anatase enabled the consideration of the respective mass fractions for particle mass calibrations as shown in Figure 7c,d. Considering further density and assuming spherical geometries finally enabled size modeling as shown in Figure 7e,f. In the case of PS particles, a 5 μm standard was analyzed for a proof of concept and accurate mean sizes could be calibrated via OF2i-SP ICP-MS. Similarly, anatase particles were obtained from a 21 nm commercial standard; however, the analysis of anatase particles was more complex as particles are known to agglomerate depending on matrices and particle number concentrations. Furthermore, TiO_2_ is a ubiquitous contaminant that complicated an interference-free analysis. To avoid unrelated TiO_2_ signals, a blank was recorded before and after the OF2i trapping. Interfering particles during blank analysis were consistently below 100 nm, and therefore, the size trapping range was tuned to only retain particle agglomerates with sizes above 100 nm, and a manual threshold was set in SP ICP-TOFMS, accordingly.

### Future Potential and Remaining Challenges

The online coupling of OF2i with SP ICP-MS has conspicuous advantages, which enable a more comprehensive analysis of individual particles. This work demonstrated the analysis of TiO_2_ and microplastic particles, which are just two representatives of a vast range of particles used or emitted in anthropogenic pathways. In particular, plastic particles are composed of a large group of entities with very diverse (eco-)toxicological behaviors and species need to be identified for accurate calibrations and investigations of environmental impact and fate. The optical characterization of trapped particles via Raman scattering before counting discrete particles and estimating elemental composition as well as masses/sizes via SP ICP-MS has a high potential to advance the research on microplastics. OF2i may become an interesting tool for selective preconcentration and sizing of particles from complex matrices and especially TOF-technology for SP ICP-MS holds the key to investigate other isotopes/elements, which are associated with plastic particles (e.g., adsorbed heavy metals^37^).

Both OF2i and SP ICP-MS enable the estimation of particle sizes. While this may be useful for an intrinsic validation, it has utility for complementary investigations. Size detection limits for both methods follow completely different paradigms. While in OF2i, large particles with high refractive index are difficult to analyze, figures of merit in SP ICP-MS depend on the element of interest and the sample matrix setting a lower limit for investigations of small particles. As such, OF2i-SP ICP-MS can be used to cover a larger size range of particles with various physical properties and therefore detect particles that would have been missed by the individual techniques. Further, the ability to account for missed particle events and particle fractions may be an interesting feature for a size and number balancing system.

In this study, particles were trapped, optically characterized, and then released for SP ICP-TOFMS. This enabled the optical and elemental analysis of the same particles. However, given that only a limited number of particles should be trapped at the same time, a representative subsample of particles may be analyzed to inquire about relevant parameters for SP ICP-MS modeling. The number of particles trappable on the vortex beam is estimated to be around 50 entities. At higher numbers, it becomes more likely that particles collide and form agglomerates. The agglomeration of particles can become pronounced in the vortex beam due to optical effects: the scattering of light on particles is inherent in its refraction and the formation of interference patterns. Especially directly after a particle, an optical jet is formed stimulating the agglomeration of particles as explained by Šimić et al.^22^ This effect needs to be considered when particles are counted and sizes are calibrated (compare Figure 5a, white arrow). However, the modeling of mass and size distributions via SP ICP-TOFMS required the detection of a sufficient number of particles. In this study, trapping, optical characterization, and release took several minutes. This may be improved with the application of a dedicated microfluidic circuit with six-port valves and may be beneficial to accelerate trapping and release cycles and therefore to increase the counting frequency in a hyphenated setup. While it was possible to analyze the same particles via OF2i and SP ICP-TOFMS, a retrospective allocation of individual events was difficult. The gradual release (optical chromatography) of particles in conjunction with short washout/transition times may accomplish this.

## Conclusions

This work demonstrated the feasibility of coupling OF2i with Raman and SP ICP-TOFMS. These unique combinations enable harnessing the technology-specific advantages and retrieve data on a molecular and atomic level coherently. In this study, OF2i-SP Raman and OF2i-SP ICP-TOFMS experiments were conducted separately due to laser safety requirements. However, the implementation of an SP Raman module does not interfere with the coupling of OF2i-SP ICP-TOFMS and supports the unification of all three techniques in one platform (OF2i-SP RAMAN-SP ICP-TOFMS). This provides unique opportunities for comprehensive and complementary single particle characterization. OF2i enabled the trapping of particles for the elimination of interfering matrix constituents prior to SP analyses. Further, optical characterizations via OF2i-Raman accomplished the inquiry of chemical species/phases, which is a key feature to improve accuracy in SP ICP-TOFMS. The latter enabled investigations of elemental compositions in single particles and further established mass and size distribution models for two representative particle types. The combination of optical and elemental MS advances the characterization of single particles and fills current analytical gaps, allowing more in-depth and accurate analysis.

## Supporting Information Available

Further explanation and details on principles and processes in OF2i are provided; theoretical and practical details for the coupling and understanding of OF2i-Raman-SP ICP-TOFMS are given; Figure S1A shows the positioning of particles across the vortex laser beam when aligning fluidic and optical forces antiparallelly; the alignment is size-dependent and can be followed in real time by observing the Rayleigh scattering; Figure S1B shows the forward scattering and the shape of the vortex beam; Figure S2 (left) shows the Raman scattering of two trapped TiO_2_ particles; and for each of the particles, calibrated Raman spectra (b, c) were compared against the reference spectra of rutile and anatase (PDF)
