# Supplementary material for: Optofluidic Force Induction Meets Raman Spectroscopy and Inductively Coupled Plasma-Mass Spectrometry: A New Hyphenated Technique for Comprehensive and Complementary Characterizations of Single Particles
Source: Anal Chem. 2024 May 14;96(21):8291–9. doi: 10.1021/acs.analchem.3c04657 (PMC11140667; doi:10.1021/acs.analchem.3c04657)
Supplement: Supplementary file 1 — ac3c04657_si_001.pdf [file ac3c04657_si_001.pdf]

Supporting Information

Optofluidic Force Induction meets Raman Spectroscopy and Inductively Coupled Plasma – Mass Spectrometry: A new hyphenated technique for comprehensive and complementary characterizations of single particles.

Christian Neuper[1,7, ‡], Marko Šimić[1,2,3, ‡], Thomas E. Lockwood[4], Raquel Gonzalez de Vega[5], Ulrich Hohenester[3], Harald Fitzek[7], Lukas Schlatt[6], Christian Hill[1,2], David Clases[5,*]

[1] Brave Analytics GmbH, 8010 Graz, Austria
[2] Gottfried Schatz Research Center, Medical Physics and of Biophysics, Medical University of Graz, 8010 Graz, Austria
[3] Institute of Physics, University of Graz, 8010 Graz, Austria
[4] University of Technology Sydney, 2007 Ultimo, Australia
[5] Institute of Chemistry, University of Graz, 8010 Graz, 8010 Graz, Austria
[6] Nu Instruments, LL13 9XS Wrexham, United Kingdom
[7] Graz Centre for Electron Microscopy, 8010 Graz, Austria

‡ These authors contributed equally
* Corresponding author: David Clases, David.Clases@uni-graz.at.

Optofluidic force induction – considering optical and drag/fluidic forces

In previous studies, the set-up of OF2i involved the parallel alignment of fluidic and optical forces. This enabled a trapping of adjacent particles via gradient forces in the weakly focused vortex beam and a subsequent acceleration via scattering forces in the propagation direction[1]. After trapping, the particles move along the intensity maxima of the laser beam with propagation distance z and velocity v, in presence of optical scattering forces $F_{\mathrm{opt}}(z)$ and fluidic forces $F_{\mathrm{fluid}}(v)$, which for laminar flow and spherical particles are described according to equation (S1).

$$F_{\mathrm{fluid}}(v) = -6\pi\eta R(v - v_{\mathrm{fluid}}) \quad (\mathrm{S1})$$

Here η is the viscosity and v_{fluid} the velocity of the fluid, and R is the sphere radius. For particles with radii above a few tens of nanometres, the momentum relaxation time is so short that $\dot{v} \approx 0$,

and Newton's equation of motion $m\dot{v} = F_{\text{opt}}(z) + F_{\text{fluid}}(v) \approx 0$ allows calculation of the particle velocity via

$$v\,(z) = v_{\text{fluid}} + \frac{F_{\text{opt}}(z)}{6\pi\eta R} \qquad \text{(S2)}$$

As the optical force depends on R, the change of velocity can be translated into a particle size and the observation of several particles provides us with a particle size distribution[1,2]. In this work, we aligned the laser beam antiparallelly to the fluid direction such that the optical force decelerated the particle. If the magnitude of the optical force was equal to that of $F_{\text{fluid}}(0)$, the particle came to a complete halt at a stable trapping position z_{trap} where the optical force counteracted the drag force of the fluid,

$$F_{\text{opt}}(z_{\text{trap}}) + F_{\text{fluid}}(0) = 0 \qquad \text{(S3)}$$

Figure 2a (main article) shows simulated trajectories[1,2] of PS-based plastic particles with different diameters transported by the fluid in presence of the focused laser beam (propagating from right to left). It is visible that particles are first pushed towards the intensity maxima by the gradient forces, and then become trapped at size-dependent positions where they continue to orbit around the optical axis because of the orbital angular momentum of the laser beam. This momentum plays a pivotal role and gives rise to the ring-shaped intensity profile in the transverse directions. The unhindered passing of smaller particles through trapping regions is only possible with such a configuration. The minimum and maximum trappable size can be tuned by altering fluidic flow rates as well as laser intensity. In this case (Figure 2, main article), parameters were set to trap particles with diameters between 350 and 1500 nm, and particles below 350 nm continued to flow through the capillary (compare trajectory of blue particle with d=300 nm, Figure 2a).

Considerations for coupling OF2i and SP Raman

For the coupling of OF2i and SP Raman, a conventional OF2i- instrument was opened up and modified: Light scattered by trapped particles was magnified and recorded using an ultramicroscope setup with a 10x PLAN objective, an optical filtering bank, a 75 mm focusing lens, and a CMOS camera. The vortex beam and forward scattered light was recorded additionally to monitor beam shape. Figure S1 (A) shows the scattered light of individual particles, which is portraited as line due to the cylindrical lens. It is visible that particles trapped upstream (left) show a more pronounced scattering. This is due to the size-dependent position of particles and the fact that large particles show more pronounced light scattering. Large particles experience a higher level of optical forces and are therefore pushed upstream until optical and fluidic/drag

forces cancel each other out. Figure 1B shows an image parallel to the optical axis. The 2D projection of the vortex beam shows here a “donut shape” and some forward scattering and interference patterns are visible.

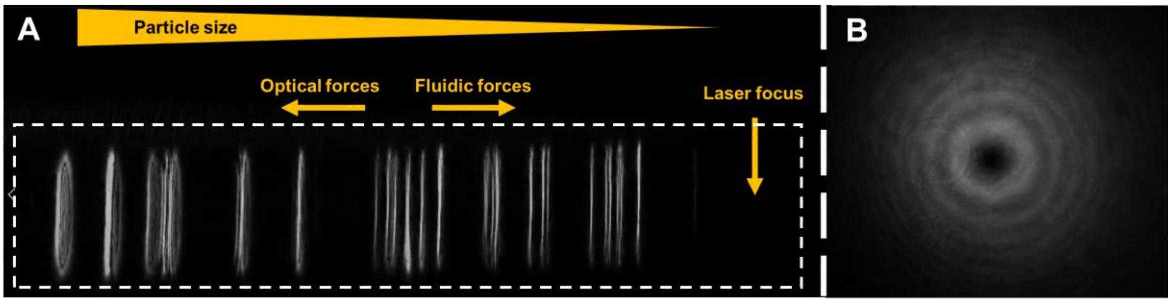

Figure S1. A: Each white line indicates one trapped particle, which are sorted according to their size along the optical axis. Stable positions are reached upstream of the laser focus where optical and drag forces cancel each other out. B: Forward projection of the vortex beam.

The scattered light was subsequently analysed using a prism to diffract and distinguish Rayleigh (elastic) and Raman (inelastic) scattering. Given that the latter is phase/species depending, spectra were recorded and compared against reference spectra to identify the particle identity. Figure S2 (top) shows the scattering of two trapped TiO_2 particles (see blue arrows). Broad white bands parallel to the z-axis correspond to the inelastic scattering of water. The Rayleigh scattering is visible at the bottom and the wavenumber shift due to Raman scattering is portraited across the y-axis. Reading out wavenumber-dependent intensities allows to plot the Raman spectrum for single particles as shown in Figure S2 b and e, which could subsequently be compared against reference spectra for common TiO_2 phases. In this case, particles consisted of anatase, which was critical information for subsequent size and mass calibrations via SP ICP-TOFMS.

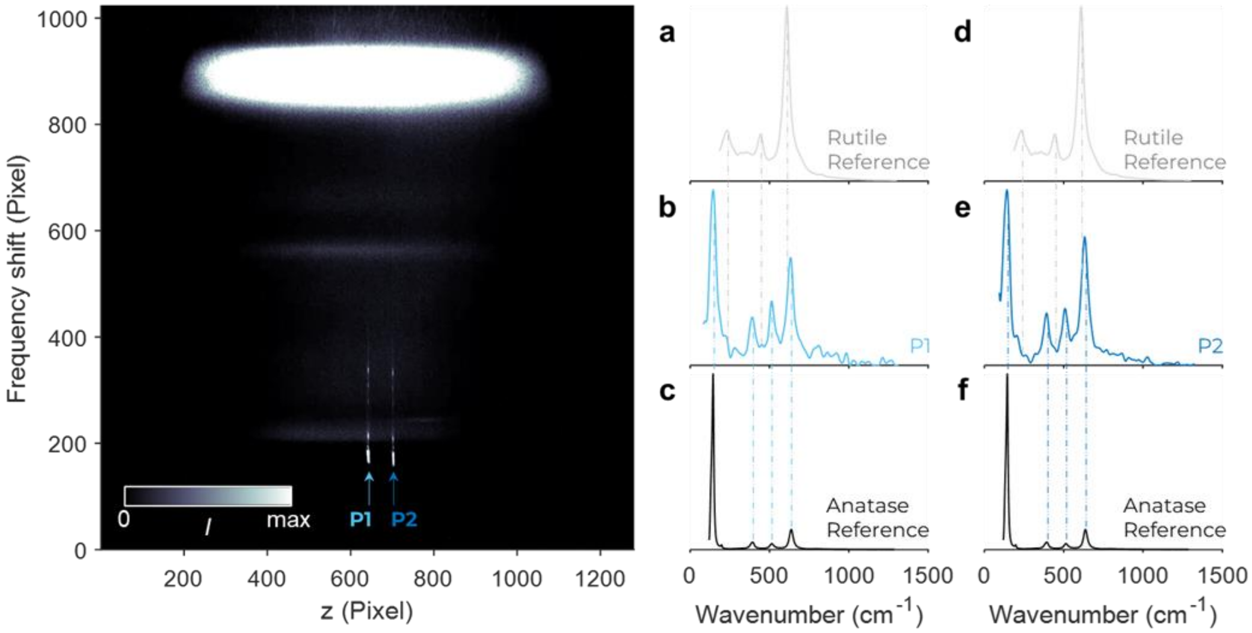

Figure S2 TiO_2 particles were trapped and analysed via OF2i-Raman (left). The experimental Raman spectra (b, e) were compared against those of anatase (c, f) and rutile (a, d) as common TiO_2 phases enabling a clear identification of the former.

Coupling OF2i and SP ICP-TOFMS

The flow rates employed as well as the SP counting frequency of both OF2i and SP ICP-TOFMS are comparable which are in the lower to mid microliter/minute range. This facilitated the coupling substantially: A transfer capillary was attached to the outlet of the OF2i-cell and attached via PEEK adapters to the SC-kit enabling direct transfer. In the presented set-up, several dead volumes, a turbulent flow as well as adsorption could not be avoided leading to rather long wash out times and delays as well as a statistical mixing of particles released in OF2i. While it was possible to follow packages of particles released by OF2i, it was not possible to pinpoint and follow individual particles between the two techniques. This may be overcome by developing a more dedicated transfer system. It was further observed that the particle loading step could contaminate the transfer line and adsorbed particles could randomly be released – even during the actual particle release step. This was a problem specifically for TiO_2 particles and required long washing times to reduce the number of random TiO_2 events. In the future, a dedicated six-port-valve system may be adequate to decouple the loading step and to bypass the SP ICP-TOFMS system to avoid adsorption in transfer lines and retention in dead volumes.

OF2i can be operated in two modes: flow mode, trapping mode (see above and compare Figure 3 (main article)). The trapping mode is specifically interesting when aiming to retrieve species-specific data via SP Raman spectroscopy and can be coupled online with SP ICP-TOFMS. In cases

where abundant particles are analysed and it is not important to analyse the exact same particles trapped previously via OF2i, conventional concentric nebulisers and spray chambers are adequate despite their low transport efficiency ranging typically between 1 and 5%. However, in cases where rare particles are to be analysed, higher transport efficiency is desirable. In this work, it was one objective to showcase the possibility to trap particles via OF2i to exchange matrix as well as to perform optical characterisation before detecting the same particle via SP ICP-TOFMS. The maximum number of trappable particles for the suggested system is about 50 and leads to the dilemma that only a fraction of particles can be recovered with a conventional nebuliser system. Therefore, a nebuliser system with a higher transport efficiency was inquired instead. A SC introduction kit (Elemental Scientific, Omaha, US) was employed here and enabled an efficiency of 66%.

References

(1) Šimić, M.; Auer, D.; Neuper, C.; Šimić, N.; Prossliner, G.; Prassl, R.; Hill, C. & Hohenester, U. Real-time nanoparticle characterization through optofluidic force induction. *Phys. Rev. Appl.* **2022**, *18(2)*, 024056.

(2) Šimić, M.; Hill, C.; & Hohenester, U. Theoretical description of optofluidic force induction. *Phys. Rev. Appl.* **2023**, *19(3)*, 034041.
